# Localized Response of De Novo Terpenoid Emissions Through the Jasmonate Signaling Cascade in Two Main European Tree Species

**DOI:** 10.1111/ppl.70432

**Published:** 2025-07-31

**Authors:** Mirjam Meischner, Simon Haberstroh, Jürgen Kreuzwieser, Baris Weber, Andrea Ghirardo, Jörg‐Peter Schnitzler, Christiane Werner

**Affiliations:** ^1^ Ecosystem Physiology University of Freiburg Freiburg Germany; ^2^ Research Unit Environmental Simulation Helmholtz Zentrum München Neuherberg Germany

**Keywords:** herbivore induced plant volatiles (HIPVs), roots, terpenoid storage pools, volatile organic compounds, within‐plant signaling

## Abstract

The systemically induced production of volatile organic compounds (VOCs) in undamaged tissues of plants under herbivore attack is still not fully understood, particularly with respect to below‐ and aboveground signaling. Here, we test the hypotheses that treatment of trees with jasmonic acid (JA) to simulate local herbivory (i) systemically induces VOC emissions in leaves and roots by signal propagation via the vascular bundle system and (ii) that bidirectional signaling occurs between below‐ and aboveground organs. We applied JA to roots and branches of *Fagus sylvatica* and *Picea abies* in a controlled experiment and shielded untreated tissues from volatile cues. VOC emissions and gas exchange were measured continuously over 6–8 days and complemented by quantification of tissue terpenoid storage pools. In contrast to the strong increase in terpenoid emissions from directly treated leaves and needles, which were mainly composed of sesquiterpenes, no systemically induced terpenoid emissions were found. Direct JA treatment of shoots reduced net photosynthesis and stomatal conductance in *P. abies* by ~50%, while the gas exchange of *F. sylvatica* remained unaffected. In the root system of *P. abies* , terpenoid contents increased both locally and systemically in response to belowground JA treatment. Overall, our results challenge the concept of systemically induced terpenoid emissions through vascular JA signaling, which is commonly induced in trees in response to insect herbivory. Instead, our data point toward a possible role of volatile cues in intra‐plant signaling.

## 1 Introduction

Herbivore‐induced defenses are key adaptations of sessile plants to resist pathogenic insects and microorganisms and involve various changes in plant morphology and metabolism (Karban and Myers 1989; Howe and Jander 2008). These include, but are not limited to, the strengthening of cell walls (Hückelhoven 2007), the formation of traumatic resin ducts (Martin et al. 2002; Miller et al. 2005) and, importantly, the biosynthesis of proteins and secondary metabolites that have a repellent effect on phytophagous insects due to their poor digestibility or toxicity (Rosenthal et al. 1979; Edwards et al. 1993; Mithöfer and Boland 2012; Bertić et al. 2023). Among the chemically diverse group of secondary metabolites, volatile organic compounds (VOCs) contribute to direct defenses, as well as to indirect plant defenses by mediating plant–plant, plant–insect, and multitrophic interactions, such as attracting predatory insects (Turlings and Tumlinson 1992; Dicke 1994). Plant defense responses can be induced locally at the site of attack or systemically in (as yet) unaffected parts of the plant, making them more resistant to future insect attack (Green and Ryan 1972; Karban and Myers 1989; Bostock 2005). The majority of studies in the field of induced systemic resistance (ISR) have focused on herbaceous (model) species, such as *Arabidopsis thaliana* and *Nicotiana tabacum* (reviewed by Gatehouse 2002; Karban and Baldwin 2007), but much less is known about the responses of woody plants (reviewed by Eyles et al. 2010). Woody plants, such as trees, have evolved different defense strategies, including the formation of specialized resin ducts to store terpenoids in the tissue of conifers (Ghirardo et al. 2010; Niinemets et al. 2011), in contrast to the labile terpenoid pools found in many deciduous broadleaved trees (Dindorf et al. 2006; Holzke et al. 2006). For both conifers and broadleaved species, it has been demonstrated that insect herbivory can systemically induce VOC emissions (Eyles et al. 2010). For example, in experiments on hybrid poplars, caterpillars of the moth *Malacosoma disstria* systemically induced the emission of the sesquiterpene (−)‐germacene D from leaves (Arimura et al. 2004), and the stem‐boring white pine weevil (*Pissodes strobi*) led to systemically induced terpene emissions from undamaged foliage of Scots pine ( *Pinus sylvestris* ), including emissions of linalool, β‐phellandrene, limonene, and 1,8‐cineole (Heijari et al. 2011). It is noteworthy that the systemic induction of defense responses is not limited to the attacked organ (i.e., leaves, stem or roots), but the concentrations of secondary metabolites in the foliage can also be induced by root herbivores (Kaplan et al. 2008; Tytgat et al. 2013). For example, the feeding of *Agriotes lineatus* on roots of cotton plants ( *Gossypium herbaceum* ) increased concentrations of terpenoid aldehydes, such as hemigossypolone, in leaves (Bezemer et al. 2004; Bezemer and van Dam 2005), suggesting a link between induced root and leaf defenses that remains poorly understood (Erb et al. 2009).

An important plant hormone mediating the defense response between different parts of the plant is jasmonic acid (JA) (Wasternack and Hause 2002; Wasternack 2007). Its exogenous application is commonly used to mimic plant responses to herbivory (Hopke et al. 1994; Martin et al. 2003; Ballhorn et al. 2008; Tytgat et al. 2013) as it allows highly standardized experiments (Waterman et al. 2019) and induces similar VOC emissions as real insect herbivores (Degenhardt and Lincoln 2006), except for the release of VOCs due to wounding, for example, green leaf volatiles (Li et al. 2019). JA functions to propagate information about the presence of a local insect attack throughout the plant (Thorpe et al. 2007) and to upregulate the expression of defense‐related genes, including terpene synthases (Martin et al. 2003; Zhou et al. 2020). Activation of the JA pathway can thus lead to increased emissions of mono‐ and sesquiterpenes (Boland et al. 1995; Martin et al. 2003; Volf et al. 2021) and to the accumulation of semi‐volatile diterpenes in storage pools (Martin et al. 2002). Terpenes and oxygenated terpenes (= terpenoids) are derived from a five‐carbon precursor (Dudareva et al. 2013) and are integral components of the induced chemical defense system of trees against insects due to their function as signaling molecules and their repellent effect on many insects (Unsicker et al. 2009).

Currently, it is widely recognized that woody species respond differently to herbivory than herbaceous species, but detailed characterizations of the induced resistance of many woody species are still lacking, especially considering the potentially different responses of species with and without permanent terpenoid storage pools. The experimental approach to analyze the ISR of plants generally includes two ways of signal transduction pathways: (i) the transport of phytohormones via the vascular bundle system and (ii) airborne signaling by stress‐induced VOCs (Engelberth et al. 2004; Frost et al. 2007). Experiments disentangling both pathways are needed to improve our process understanding of ISR in trees. Furthermore, it remains unclear how the reaction of shoots and roots differs and whether or not belowground herbivory induces significant aboveground terpenoid emissions from the shoot and, conversely, to what extent shoot herbivory induces root defenses.

In this study, we test the hypotheses that (i) terpenoid emissions are systemically inducible in broadleaved and coniferous trees, excluding airborne signals, and that (ii) root‐shoot signaling can lead to terpenoid production in the shoot and *vice versa*. To test our hypotheses, we simulated herbivory on two dominant Central European tree species: *Picea abies* L. (Karst) (Norway spruce), an evergreen coniferous species characterized by the presence of resin ducts and high terpenoid contents, and *Fagus sylvatica* L. (European beech), a deciduous broadleaved species lacking specialized terpenoid storage pools. Herbivory was simulated by applying JA, either to the shoot or the roots of 3‐year‐old saplings under controlled conditions. Importantly, untreated tissues were shielded from stress‐induced VOCs emitted from directly treated parts of the plant. The dynamics of VOC emissions and gas exchange of treated and untreated branches and roots were measured continuously over 6–8 days in combination with the quantification of stored terpenoids in needles and roots before and after the treatment with JA.

## 2 Materials and Methods

### 2.1 Plant Material

For this study, 12 *P. abies* L. (Karst) and 12 *F. sylvatica* L. saplings from a local tree nursery were grown in 5 L pots (60 vol% Floradur, 40 vol% sand, and 5 mg L^−1^ NPK fertilizer) outdoors in Freiburg (48°00′49.1″ N, 7°49′59.1″ E, Germany) from December 2022 to February 2023 and then in a greenhouse for another 2–3 months. *P. abies* and *F. sylvatica* were 3 years old at the beginning of the experiment and had a mean height of 48.7 ± 1.1 cm and 83.2 ± 2.2 cm, respectively. In April 2023, *P. abies* saplings were transferred to a walk‐in climate chamber (Thermotec) and acclimatized to air temperatures of 23°C/15°C (day/night), 800 μmol m^−2^ s^−1^ photosynthetic photon flux density (PPFD) at canopy level, a relative humidity of 60%, and a day length of 12 h. *F. sylvatica* saplings were transferred to the climate chamber after leaf flushing and maturation in May 2023, 2 weeks before measurements started under the same environmental conditions as measurements of *P. abies* .

### 2.2 Experimental Setup

In order to continuously measure VOC, CO_2_, and H_2_O fluxes above‐ and belowground, trees were equipped with branch and root cuvettes made of borosilicate glass (780 and 190 mL volume, respectively). Root cuvettes were installed by exposing a bundle of roots and carefully shaking off the soil. Afterwards, the root bundle was positioned in a glass cuvette, which was then filled with glass beads (cleaned in an ultrasonic bath), sealed, and shaded with aluminum foil (see Meischner et al. 2024 for further details). The roots in the cuvettes were irrigated every second day. All root and branch cuvettes were connected to a fully automated gas flow‐through measurement system that was installed inside the climate chambers (adapted from Werner et al. 2020; Meischner et al. 2024). The measurement system consisted of a zero‐air generator (custom‐built) and mass flow controllers (OMEGA Engineering and Alicat Scientific, respectively) to regulate the flow through the cuvettes to 500 mL min^−1^, multi‐position valves (VICI‐Valco) to switch between 14 cuvettes (12 plant positions and 2 blanks) and an analyzer unit. This included a PTR‐TOF‐MS 4000 ultra (Ionicon Analytic) for VOC detection, a CO_2_‐Spectroscope (Delta Ray IRIS, Thermo Fisher Scientific) and a water vapor and CO_2_ analyzer (LI‐850, LI‐COR Environmental).

**FIGURE 1:**
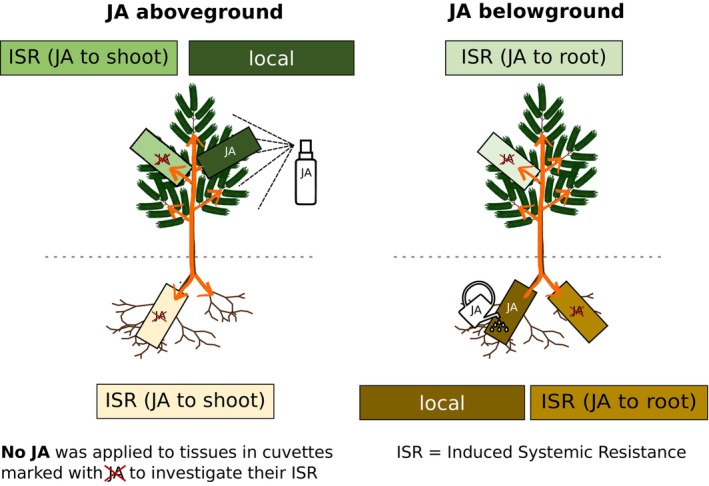
Experimental set‐up. The local and induced systemic resistance (ISR) in response to exogenous jasmonic acid (JA) application (5 mM) was analyzed in a controlled pot‐experiment on 3‐year‐old *Picea abies* (depicted) and *Fagus sylvatica* saplings. Plants were either treated with JA aboveground (shoots) or belowground (roots). The VOC emissions and gas exchange parameters (net photosynthesis, stomatal conductance, root respiration) were measured in real‐time over a time span of 6–8 days (3 days before and 3–5 days after JA application). For this, glass cuvettes (indicated as rectangles) were connected to an automated measurement system in two walk‐in climate chambers. A total of six replicates were analyzed for each of the two tree species and treatments.

Prior to the measurements, the trees were divided into two groups: half of the trees were assigned to an aboveground stress treatment (*n* = 6 per species) and equipped with two branch and one root cuvettes (Figure 1). The other half of the trees were assigned to a belowground stress treatment (*n* = 6 per species) and accordingly equipped with one branch and two root cuvettes. On day zero, after 2 days of control measurements, stress was induced both locally and systemically by application of JA either above‐ or belowground. For the aboveground JA treatment, 2 mL of a 5 mM JA solution (JA dissolved in 5 vol% ethanol, method adapted from Thaler et al. 1996) was sprayed evenly over the entire shoot, including the branch inside one of the two branch cuvettes. No droplets were observed to rinse down the plants; thus, the treatment corresponds to 0.01 mmol of JA applied per plant. The use of ethanol as a solvent for JA could possibly have a minor influence on the release of VOCs; however, it has been shown elsewhere that the stress response is primarily induced by the jasmonate (Li et al. 2019). We also tested the effect of a 5 vol% ethanol solution on the photosynthesis of an additional six *P. abies* saplings, but found no significant effect (data not shown). Simulating herbivory by exogenous application of JA has the advantage that both tree species were treated identically and the effect of activating the JA signaling cascade could be studied separately from other herbivory‐induced stimuli (Waterman et al. 2019). This also implies that mechanical stimuli and wounding by herbivory insects are not covered by this method.

The other enclosed branch remained untreated, as were the roots of the same trees. This allowed the response of locally treated branches (local JA) to be compared with the ISR of untreated branches and roots. As both the locally treated and untreated branches were inside branch cuvettes and supplied with purified air, they were shielded from volatile cues originating from other branches of the same plant or neighboring plants.

For the belowground JA treatment, the enclosures of roots to be treated were disconnected from the measuring system, and 2 mL of the same 5 mM JA solution was applied to the roots with a pipette through the open inlet of the enclosure. The JA solution was carefully rinsed down the root bundle and collected at the bottom of the root enclosure, where it was accessible to the fine roots. This process directly exposed the roots, particularly the root tips, to the JA solution. In this way, one of the two root cuvettes was treated with JA to compare the response of locally treated roots with the ISR of untreated roots and branches. In order to directly compare the effects of above‐ and belowground JA application on plant defense responses, the same dosage of JA was applied to the roots and shoots. To avoid contamination of the PTR‐TOF‐MS by JA, the measurements were paused for 2 h during JA application and resumed only after the solution had completely evaporated from the branches or the root cuvettes had been rinsed with 1 L of water. Measurements were then continued for a further 3–5 days after JA treatment in order to characterize the local and ISR of the trees to the stress treatment. In the first three runs (May 4–31, 2023), all *P. abies* were studied, followed by another three runs (June 1–29, 2023), where all *F. sylvatica* trees were measured. The number of plants in the above‐ and belowground stress treatments was balanced in each run.

### 2.3 Data Acquisition and Processing

The PTR‐TOF‐MS was operated in H_3_O^+^ ionization mode and further operating conditions were set to a drift tube temperature of 80°C, a drift pressure of 2.7 mbar, and a drift voltage of 503 V, resulting in an *E*/*N* of 128 Td, where *E* is the electric field strength affecting the drift tube and *N* is the number density of the drift tube buffer gas molecules. Raw data of the PTR‐TOF‐MS were recorded with IoniTOF software (version 4.4.69, Ionicon) in a measuring interval of 20 s and processed with IDA software (version 2.2.0.7, Ionicon). In a targeted approach, measurements of isoprene (detected at *m*/*z* 69.06), monoterpenes (*m*/*z* 137.12), oxygenated monoterpenes (*m*/*z* 155.13), sesquiterpenes (*m*/*z* 205.20), and oxygenated sesquiterpenes (*m*/*z* 223.21) were exported from the IDA software. The compounds present in the calibration gas (all except oxygenated sesquiterpenes) were exported as cps values and calibrated directly by dividing the cps values by the sensitivity (cps/ppb) of the instrument for these compounds. The sensitivity was obtained by measuring a multi‐component gas mixture (Apel Riemer Environmental) over different humidity steps using a liquid calibration unit (Ionicon Analytic). The ppb values of oxygenated sesquiterpenes were determined using the quantification module in IDA software: ppb values were calculated from the measured cps values based on (a) saved transmission rates which were obtained by the calibration procedure described above, (b) instrumental parameters that are relevant for quantification, such as the drift tube voltage, pressure, and temperature, and (c) reaction rate coefficients (*k*‐rates) of the detected compounds (*k*‐rate = 3.6 for oxygenated sesquiterpenes), which are directly proportional to the sensitivity of the instrument (Sekimoto et al. 2017). All further data processing and statistical analyses were performed with the software R (version 4.2.1, R Core Team 2021). First, each six‐minute measurement period of VOCs, CO_2_, and H_2_O was summarized by taking the average of the time span (the first 2 min of each measurement were discarded). The exact background for each data point was determined by interpolation between the measurements of the empty cuvettes (blanks) and then subtracted. Afterwards, VOC emission rates (nmol g^−1^ h^−1^), net photosynthesis rates *A* (μmol m^−2^ s^−1^), stomatal conductance for water vapor Gs (mmol m^−2^ s^−1^) and root respiration rates *R* (nmol g^−1^ s^−1^) were calculated (Methods S1 in Supporting Information). Fresh weight of leaves/needles or roots was used for calculations of VOC emission rates and *R*, while leaf area was used to calculate *A* and Gs. The continuous data were further aggregated by taking daily averages for visualization and statistical analysis. Alluvial plots were created to visualize the VOC emission rates using the R package ggalluvial (version 0.12.5, Brunson 2020). In this way, total emissions of terpenoids, as well as the course of individual terpenoids over the time span of the experiment, are shown.

### 2.4 Analysis of Terpenoid Storage Pools

To analyze the effects of local and systemic induced stress on terpenoid storage pools, samples of leaves/needles and roots (approximately 50 mg fresh weight) were taken 4 days before JA application as a control and 2 days after JA application. For the second sampling, the cuvettes of the indirectly treated leaves/needles and root cuvettes were opened to sample plant material. The reduced leaf/needle area inside the cuvettes after sampling was considered when calculating the gas‐exchange parameters and VOC emission rates. In addition, the VOC emission data for that day were excluded from the data set to remove potential artifacts, for example, in *P. abies* , terpenoid emissions caused by mechanical disturbance of the resin ducts (Ghirardo et al. 2010; Niinemets et al. 2011). The collected plant material was immediately shock‐frozen in liquid‐N_2_ and then ground by pestle and mortar to a homogenized powder and stored at −80°C. Endogenous terpenoid analysis followed a method adapted from our established procedure (Ghirardo et al. 2010; Clancy et al. 2016; Vanhatalo et al. 2018; Birami et al. 2021). For extraction, 200 μL of hexane (SupraSolv, Merck Chemicals GmbH) containing 859.3 pmol μL^−1^ δ‐2‐carene as an internal standard was added to 20 mg of needle powder or 50 mg of leaf/root powder. After 1 h of incubation in darkness and at room temperature, the sample was centrifuged, and 150 μL of the supernatant was recovered into GC vials. The pellet was re‐extracted with an additional 50 μL hexane for 30 min, and both supernatants were combined together. The terpenoid extracts were stored at 4°C until thermo‐desorption gas‐chromatography mass‐spectrometry (TD–GC–MS) analysis (see details in Methods S2 in Supporting Information).

Analysis of GC–MS data was performed using MassHunter Quantitative Analysis software (version 10.2, Agilent Technologies), with compound identification based on a mass spectra library (NIST Mass Spectral Library 2017). Data were processed as described in Ghirardo et al. (2020). Compounds that were not contained in the standard mixture used for calibration were approximately quantified based on the calibration factor of α‐pinene.

### 2.5 Statistical Analysis

Terpenoid emission rates and contents of stored terpenoids, as well as gas exchange parameters (*A*, Gs, *R*), were statistically analyzed for effects of the JA treatment using paired *t*‐tests between control and the three JA treatments “local JA,” “ISR (JA to shoot)” and “ISR (JA to root),” respectively. The mean terpenoid emission rates of day zero (7–10 a.m., i.e., before JA application started) and the day after JA treatment (from 7 to 10 a.m. for comparability) were used for statistical evaluation. The analysis was performed separately for each compound and tree species. Paired *t*‐tests were also used to statistically analyze gas exchange parameters by comparing the mean values of individual days and plant individuals served as the pairing variable. Resulting *p*‐values were then used to generate comparison letters indicating similarities and differences between days.

For each treatment and species, mean emission rates, terpenoid contents, and gas exchange parameters (±standard errors), as well as estimates, *t*‐values, and *p*‐values of the paired *t*‐tests are reported (Tables S1–S3). Finally, the Pearson's correlation coefficient was determined for all compounds of the terpenoid storage pools of *P. abies* roots.

## 3 Results

### 3.1 Gas Exchange

**FIGURE 2:**
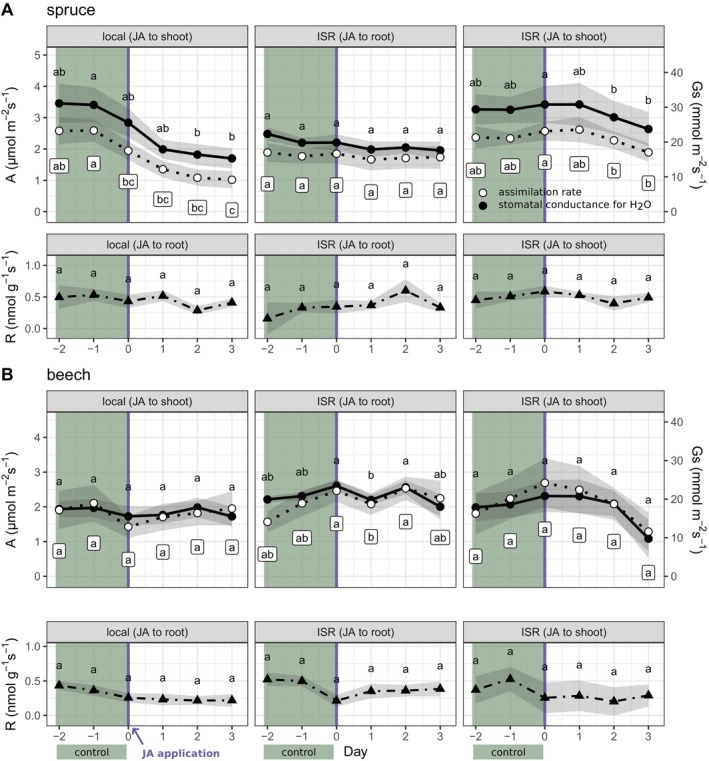
Effects of JA application on leaf/needle and root gas exchange. Branch net photosynthetic rates (*A*), stomatal conductance (Gs) and root respiration rates (*R*) were calculated from cuvette measurements of CO_2_ and H_2_O concentrations in a controlled climate chamber experiment. To investigate the signal propagation after a local stress, JA solution was applied either to the shoot or to the roots of 3‐year‐old *Picea abies* (spruce, panel A) and *Fagus sylvatica* (beech, panel B) trees (*n* = 6, each) and the local and induced systemic resistance (ISR) was analyzed in separate glass cuvettes. Daily mean values of the light phase ± standard error are shown. Comparison letters indicating similarities and differences between days were generated with paired *t*‐tests, using plant individuals as pairing variable and a significance level of *p* < 0.05.

In *P. abies* , the net photosynthetic rate (*A*) of directly treated branches decreased by half from 2.6 ± 0.4 to 1.3 ± 0.3 μmol m^−2^ s^−1^ within one day after JA application (Figure 2) and remained significantly reduced until the end of the experiment (*p* < 0.001***, Figure 2, Table S1). A similar decrease was observed for stomatal conductance (Gs), which was also significantly reduced on day three compared to the control phase (*p* = 0.002**, Figure 2, Table S1). After three days, *A* and Gs of untreated branches also decreased slightly, though not significantly, compared to the control phase from 2.3 ± 0.2 to 1.9 ± 0.3 μmol m^−2^ s^−1^ (*p* = 0.07, Figure 2, Table S1) and from 29.3 ± 3.8 to 23.7 ± 4.9 mmol m^−2^ s^−1^ (*p* = 0.08, Figure 2, Table S1), respectively. In three *P. abies* individuals, gas exchange was measured for five instead of three days after aboveground JA treatment. These measurements show that *A* and Gs did not decrease further after three days in both treatments (local and ISR), but remained at the same level (Figure S1).

In *F. sylvatica* , direct JA application did not induce stomatal closure, and *A* remained constant between 1.5 and 2.5 μmol m^−2^ s^−1^ throughout the experiment. The indirectly treated leaves of *F. sylvatica* (JA to shoot) showed a non‐significant reduction in *A* and Gs on the third day; however, the absolute values were still within the range of the control measurements (Figure 2B). The application of JA belowground had no effect on photosynthetic gas exchange in either species (Figure 2). Additionally, root respiration rates (*R*) were similar between *P. abies* and *F. sylvatica* , ranging from 0.3 to 0.6 nmol g^−1^ s^−1^, and no statistically significant decline could be observed for any treatment or species (Figure 2, Table S1).

### 3.2 Terpenoid Emissions

**FIGURE 3:**
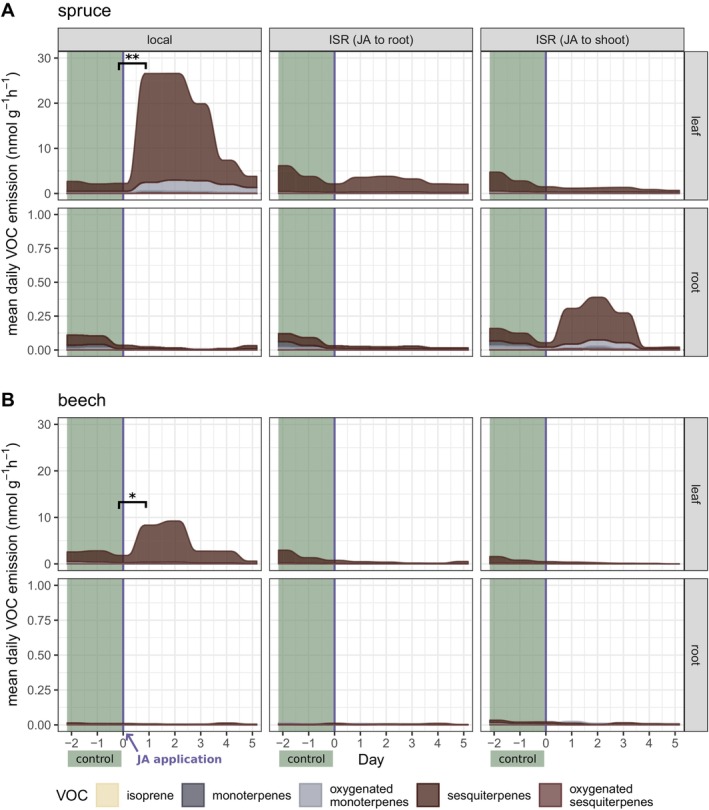
Mean diurnal terpenoid emission rates from *Picea abies* (spruce, panel A) and *Fagus sylvatica* (beech, panel B) in response to jasmonic acid (JA) application. JA was applied on day zero either to the shoot or to the root; the local and the induced systemic resistance (ISR) of leaves/needles and roots of the same plant individuals were analyzed (*n* = 6 per treatment and species). Total terpenoid emissions from day zero (7–10 a.m., i.e., before JA application started) and day 1 (7–10 a.m., for comparability) were statistically evaluated with pairwise *t*‐tests and significant differences are marked with asterisks. Significance levels of *p < 0.05, **p < 0.01, and ***p < 0.001 were used.

Total terpenoid emissions from the shoots of *P. abies* and *F. sylvatica* increased significantly (*p* = 0.010** and *p* = 0.020*, respectively) one day after JA was applied directly to needles and leaves (Figure 3, Table S2). Mainly, sesquiterpenes contributed to the increase in total terpenoid emissions in both *P. abies* and *F. sylvatica* due to their high emission rates compared to other compounds (up to 26 and 10 nmol g^−1^ h^−1^, respectively). In *P. abies* , emissions of isoprene (*p* = 0.002**), oxygenated monoterpenes (*p* = 0.048*) and oxygenated sesquiterpenes (*p* = 0.015*) also increased significantly (Table S1) compared to the constitutive emissions before JA application. Only monoterpene emissions were not affected by the local JA application (Figure 3A, Table S2). In *F. sylvatica* , the emissions of oxygenated sesquiterpenes also increased significantly compared to controls (*p* = 0.014*), whereas the emissions of monoterpenes and oxygenated monoterpenes remained at pre‐stress levels (Figure 3B). No change in terpenoid emissions was found for indirectly treated branches for both species (Figure 3, Table S2).

Interestingly, two out of six *P. abies* individuals showed an increase in mono‐ and sesquiterpene emissions from the roots in response to aboveground JA application, as reflected in the mean emission rates of this treatment (Figure 3A). Although this effect was not significant for total terpenoid emissions, most likely due to the highly variable response of plant individuals to the JA treatment, the emission rate of monoterpenes from the roots in response to aboveground JA application doubled significantly from 0.002 to 0.005 nmol g^−1^ h^−1^ (*p* = 0.041*) (Table S2B). No other effects in root emission were found for any species, and belowground JA application had no effect on total terpenoid emissions (Figure 3). In *P. abies* , there was only a marginal increase in mean sesquiterpene emissions from needles the day after belowground JA application (Figure 3, Table S2).

### 3.3 Terpenoid Contents

**FIGURE 4:**
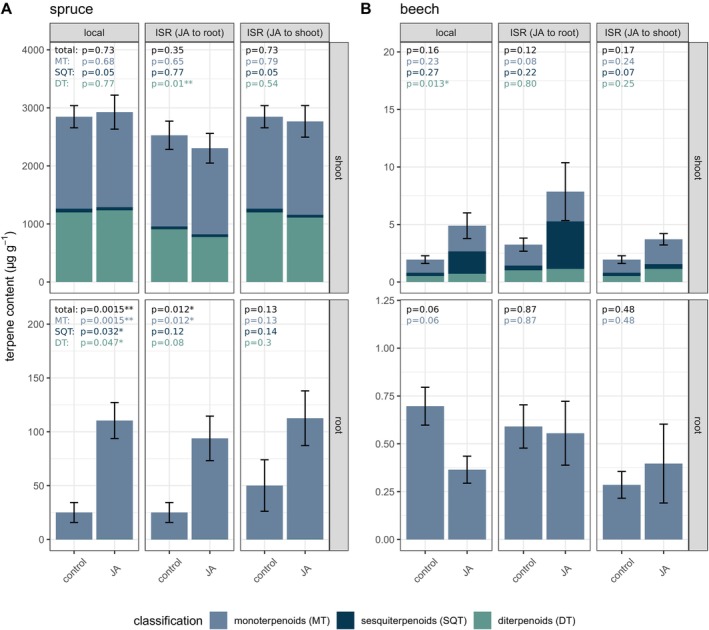
Terpenoid content in roots and needles/leaves of *Picea abies* and *Fagus sylvatica* in response to jasmonic acid (JA) treatment (*n* = 3–6). JA was applied on day zero of the experiment either to the shoot or to the roots. Controls were collected 4 days prior to the application of JA and were employed for pairwise *t*‐tests with local and induced systemic resistance (ISR) samples collected 2 days after JA application from the same plant individuals. For statistical analysis, total terpenoid contents were used, as well as sums of mono‐, sesqui‐, and diterpenes, and significance levels of *p < 0.05, **p < 0.01 and ***p < 0.001 were applied. Note the difference scales in (A) and (B) for the different species and plant organs. DT, diterpenes; MT, monoterpenes; SQT, sesquiterpenes.

In *P. abies* , direct treatment of needles with JA did not lead to an increase in the total content of stored terpenoids compared to the control measurements, in contrast to the observed increase in stress‐induced terpenoid emissions (Figure 4A, Table S3). Neither did the content of individual terpenoids in the needles increase in response to direct JA application (Table S3). Also, the composition of terpenoid contents and emissions differed. While stress‐induced terpenoid emissions were dominated by sesquiterpenes, they represented only a small fraction of the entire terpenoid content (about 2.3%) of the needles, which was relatively balanced between mono‐ and (semi‐volatile) diterpenoids. The two compounds with the highest concentrations in the needles were identified as bornyl acetate, which accounted for approximately 17% of the total terpenoid content, and a diterpene tentatively identified as 13‐epimanool, which contributed 14%. In *F. sylvatica* , application of JA to the shoot tended to increase the terpenoid content of the leaves as both a locally and systemically induced response, although this effect was statistically not significant. Also, belowground JA treatment led to a weak, though non‐significant increase in sesquiterpene content (from 0.41 ± 0.08 to 4.13 ± 2.69 μg g^−1^; *p* = 0.22) in the leaves of *F. sylvatica* (Figure 4B, Table S3).

**FIGURE 5:**
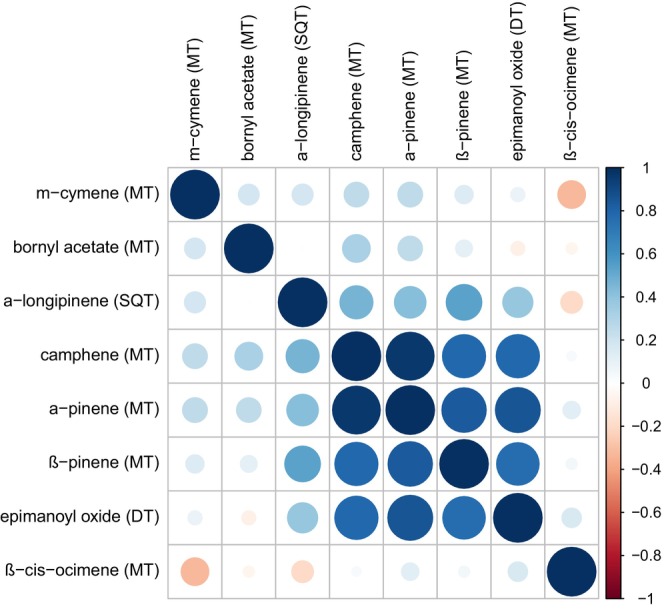
Correlation matrix with Pearson's correlation coefficients of all compounds identified in the roots of *Picea abies* with GC–MS analysis. DT, diterpenes; MT, monoterpenes; SQT, sesquiterpenes.

When *P. abies* saplings were treated belowground with JA (Figure 1), there was a clear increase in the total terpenoid content of roots (*p* = 0.001**, Figure 4, Table S3) as a local response to JA application and also as a systemically induced response (*p* = 0.012*, Figure 4, Table S3). More specifically, monoterpenes with the highest concentrations (i.e., α‐pinene and β‐pinene) as well as less concentrated compounds (such as camphene, the sesquiterpene α‐longipinene and a diterpene tentatively assigned as epimanoyl oxide) were induced by JA. Except for α‐longipinene, all these compounds were highly correlated (Figure 5), indicating synthesis by the same multi‐product enzyme. After shoot JA treatment, the total terpenoid content in roots increased on average, but not significantly (*p* = 0.13, Figure 4A, Table 1), reflecting the observed increase in terpene emissions from roots (Figure 3A). Besides this, the composition of the terpenoid content in the roots of *P. abies* did not reflect that of emissions, as the terpenoid emissions from the roots were composed of 72% by sesquiterpenes and only 11% by monoterpenes under control conditions (Table S2B), while the root terpenoid content was dominated by monoterpenes.

**TABLE 1:** Terpenoid content in roots of *Picea abies* .

Terpene content in spruce roots						
Compound name	Chemical class	Control versus local (*n* = 6)				
		*Control*	Local	Estimate	*T*	*p*
α‐pinene	MT	8.32 ± 3.43	44.93 ± 7.78	36.62	5.72	0.002**
Camphene	MT	3.13 ± 1.12	15.14 ± 2.16	12.01	5.50	0.003**
m‐cymene	MT	0.00 ± 0.00	0.14 ± 0.10	0.14	1.41	0.218
β‐pinene	MT	9.82 ± 4.83	43.86 ± 9.49	34.05	4.33	0.008**
β‐cis‐ocimene	MT	3.57 ± 2.07	4.25 ± 2.02	0.68	0.40	0.709
Bornyl acetate	MT	0.15 ± 0.05	1.61 ± 0.80	1.46	1.88	0.118
α‐longipinene	SQT	0.04 ± 0.02	0.13 ± 0.06	0.10	2.96	0.032*
Epimanoyl oxide	DT	0.06 ± 0.03	0.39 ± 0.15	0.33	2.63	0.047*
Total terpenes		25.08 ± 9.24	110.45 ± 16.70	85.38	6.31	0.001**

Compound name	Chemical class	Control versus ISR (JA to root) (*n* = 6)				
		*Control*	ISR (JA to root)	Estimate	*T*	*p*
α‐pinene	MT	8.32 ± 3.43	35.84 ± 9.72	27.53	2.76	0.040*
Camphene	MT	3.13 ± 1.12	11.91 ± 3.11	8.79	3.02	0.029*
m‐cymene	MT	0.00 ± 0.00	0.00 ± 0.00	0.00	1.06	0.338
β‐pinene	MT	9.82 ± 4.83	42.21 ± 10.37	32.39	3.99	0.010*
β‐cis‐ocimene	MT	3.57 ± 2.07	3.17 ± 1.50	−0.40	−0.17	0.868
Bornyl acetate	MT	0.15 ± 0.05	0.25 ± 0.13	0.10	0.75	0.488
α‐longipinene	SQT	0.04 ± 0.02	0.12 ± 0.07	0.09	1.89	0.117
Epimanoyl oxide	DT	0.06 ± 0.03	0.40 ± 0.16	0.34	2.17	0.082
Total terpenes		25.08 ± 9.24	93.90 ± 20.67	68.82	3.81	0.012*

Compound name	Chemical class	Control versus ISR (JA to shoot) (*n* = 6)				
		*Control*	ISR (JA to shoot)	Estimate	*T*	*p*
α‐pinene	MT	21.55 ± 11.52	46.97 ± 9.74	25.41	1.96	0.108
Camphene	MT	7.28 ± 3.75	15.60 ± 2.48	8.32	1.99	0.104
m‐cymene	MT	0.22 ± 0.17	0.34 ± 0.15	0.12	0.68	0.525
β‐pinene	MT	19.85 ± 9.31	45.07 ± 12.68	25.22	1.56	0.178
β‐cis‐ocimene	MT	0.65 ± 0.65	3.53 ± 2.24	2.88	1.54	0.184
Bornyl acetate	MT	0.38 ± 0.14	0.52 ± 0.12	0.14	0.87	0.422
α‐longipinene	SQT	0.04 ± 0.02	0.12 ± 0.04	0.08	1.78	0.136
Epimanoyl oxide	DT	0.15 ± 0.08	0.43 ± 0.13	0.29	1.79	0.133
Total terpenes		50.11 ± 23.89	112.59 ± 25.40	62.48	1.83	0.126

*Note:* Mean contents of stored terpenoids per group are given in μg g^−1^ dry weight ± standard errors. Please note that control measurements marked in italics are identical [control samples were taken before JA application and then used for pairwise comparison with the direct and induced systemic resistance (ISR) of the same plant individuals]. Estimates, *t*‐values and *p*‐values were determined with paired *t*‐tests and significance levels of *p < 0.05, **p < 0.01 and ***p < 0.001 were applied.

In *F. sylvatica* roots, the application of JA to the roots led to a non‐significant decrease in the local terpenoid content (*p* = 0.064) (Figure 4B), an inverse response compared to the increased terpenoid content in *P. abies* roots. It should be noted that due to the very low terpenoid contents in the leaves and roots of *F. sylvatica* and the relatively high variability between replicates, data on terpenoid contents of *F. sylvatica* should be interpreted with caution.

In conclusion, the direct application of JA to the shoot resulted in a decrease of *A* and Gs in *P. abies* , but not in *F. sylvatica* , and, simultaneously, to a strong increase of terpenoid emissions in both species. No increase in terpenoid emissions was observed in the ISR treatments, with the exception of slightly enhanced root terpenoid emissions following aboveground JA application in *P. abies* . Furthermore, analysis of terpenoid contents revealed no changes in *P. abies* needles after aboveground JA treatment, whereas a local and systemic increase in terpenoid contents was detected in roots following belowground JA treatment. In *F. sylvatica* , JA treatment above‐ or belowground had no significant impact on terpenoid contents in leaves and roots.

## 4 Discussion

In this study, we challenge the concept of systemically induced terpenoid emissions in response to simulated herbivory. We provide evidence that *F. sylvatica* and *P. abies* , two dominant tree species in Europe, respond locally rather than systemically to simulated herbivory on the shoots when stress signals are transmitted exclusively through the vascular system and airborne cues are excluded. As expected, the terpenoid profiles of the two species reflected their distinct capacities for terpenoid storage, characterized by large and diverse storage pools in *P. abies* tissues, in contrast to the limited storage capacity observed in *F. sylvatica* . The observed discrepancy between emitted and stored terpenoids indicates that JA‐induced emissions of terpenoids originated to a large proportion from de novo synthesis, as discussed in more detail below.

The strong increase in terpenoid emissions from locally treated leaves and needles observed in this study aligns with previous studies in which jasmonates (JA or MeJA) were applied to plant shoots, resulting in an increase of VOC emissions (Boland et al. 1995; Filella et al. 2006; Tamogami et al. 2008; Meischner et al. 2024). This is most likely due to increased de novo biosynthesis of terpenoids since the environmental conditions (e.g., light and ambient temperature) were stable over the entire measurement period and plants were not mechanically challenged, except for the insertion in the cuvettes, where a transient effect on terpenoid emission in *P. abies* needles was observed. Besides this initial handling effect, a mechanical damage would have caused a burst of VOCs from storage pools and the rapid conversion of unsaturated membrane fatty acids to green‐leaf volatiles (GLV) via the lipoxygenase (LOX) pathway (Loreto et al. 2006). Instead, we detected consistently low emissions of the GLVs detected on *m*/*z* 99.08 and *m*/*z* 101.10 before and after JA treatment and only a very slight increase in GLVs detected on *m*/*z* 83.09 (Figure S2), supporting that there was only minor cell damage or activation of the LOX pathway (Matsui and Engelberth 2022). The activation of terpene synthases (TPS) by the JA signaling cascade is a well‐established response pattern (Fäldt et al. 2003; Martin et al. 2003) and is a plausible source for enhanced terpenoid production (cf. Meischner et al. 2024). In *P. abies* , the ratio between the amount of terpenoids emitted and those contained in the needles shifted in favor of emissions under stress, indicating that newly synthesized terpenoids were preferentially emitted rather than directed into storage pools. This could be achieved, for example, by shifts in the biosynthesis site within the tissue. An upregulation of sesquiterpene synthase activities by JA is in agreement with Martin et al. (2003) and may explain the discrepancy between emitted and stored sesquiterpenes. Furthermore, the storage organs of *P. abies* may have become more permeable due to the JA treatment, resulting in higher terpenoid emissions.

Diterpenes, on the other hand, dominated the stored terpenoid pools in *P. abies* needles while their emissions remained below the limit of detection throughout the experiment, most likely due to their low volatility and high molecular weight. Unlike *P. abies* (Ghirardo et al. 2010), *F. sylvatica* does not have specialized VOC storage structures (Dindorf et al. 2006; Holzke et al. 2006) and therefore generally has negligible terpenoid contents with no significant differences between treatments in the present study.

In addition, insect herbivory and exogenous JA application have been shown to downregulate the expression of photosynthesis‐related genes (e.g., Rubisco and Rubisco activase) (Hermsmeier et al. 2001; Bilgin et al. 2010) and to induce stomatal closure (Metodiev et al. 1996) in parallel with an upregulation of TPS activity. Indeed, the stomatal conductance of *P. abies* was reduced by the direct application of JA (Figure 2A); however, the ratio of intercellular and ambient CO_2_ concentrations (Ci/Ca) increased following JA application (Figure S3). As a consequence, CO_2_ limitation in the chloroplasts is unlikely to have caused the observed decrease in photosynthesis, and nonstomatal limitation of photosynthesis is considered to be more likely. Although the reduction of net photosynthesis rate in the remaining leaf tissues is a common response to herbivory (Zhou et al. 2015), the response is highly dependent on the plant species investigated and the type of herbivory (Nabity et al. 2009). Some plant species even increase their net photosynthesis rate on the affected branches (probably to cover the higher energy demand for defenses) (Halitschke et al. 2011) or keep it stable (Peterson et al. 2004), like *F. sylvatica* in the present study. Here we show that JA treatment induces different metabolic responses in *P. abies* and *F. sylvatica* .

In the present work, we excluded the airborne signal transduction pathways, so that the stress signals could only be transmitted within the plant, that is, by transport of phytohormones via the vascular system. JA and its derivatives are phloem mobile and important long‐distance signaling molecules (Schilmiller and Howe 2005), moving top‐down along with the photoassimilates. The transport of JA through the phloem suggests efficient signal transmission from the shoot to the root (Zhang and Baldwin 1997). However, no evidence of this link between above‐ and belowground defenses was found in our study with respect to the induction of terpenoid production. Only a systemically induced increase in terpenoid content was found, but this was restricted to the root system of *P. abies* . Notably, the local and systemic increases in total terpenoids in the roots were similar (4.4 and 3.7‐fold increase compared to control, respectively), indicating an efficient signal transduction within the root system.

Experiments with ^13^C indicate that MeJA also moves in the xylem in the opposite direction to phloem flow and that dynamic exchange between phloem and xylem promotes the rapid distribution within the plant (Thorpe et al. 2007). In our experiment, untreated branches were located at mid‐plant height between treated branches above and below to receive potential signals from both directions. However, no ISR in terms of enhanced terpenoid production was detected. In addition to the transport from treated/affected tissues, JA can also accumulate in undamaged tissues as a result of de novo synthesis (Wasternack 2007). One mechanism that could have led to no change in terpenoid emissions from untreated branches in this study—if JA levels were elevated in this tissue at all—would be a rapid resynthesis of the transcriptional repressor protein JASMONATE ZIM DOMAIN (JAZ) (Chini et al. 2007; Howe and Jander 2008). The JAZ repressor protein is degraded by the JA signaling pathway, resulting in the upregulation of previously repressed genes (Chini et al. 2007). If the JAZ repressor is quickly resynthesized after the activation of the JA pathway, the expression of energy‐demanding defense processes can be blocked (Howe and Jander 2008).

Finally, it seems reasonable to suggest that the exclusion of volatile signaling pathways may be the decisive factor in explaining the absence of systemic responses from shoots compared to other studies. For example, in *P. abies* or *Populus trichocarpa × deltoides*, an increase in terpenoid emissions from intact branches on insect‐stressed plants has been documented (Arimura et al. 2004; Blande et al. 2009). However, these and similar studies (e.g., Dicke et al. 1990; Turlings and Tumlinson 1992) allowed free air exchange between treated and untreated branches and could not distinguish between vascular and airborne transmission paths. There is evidence that airborne chemical cues for within‐plant signaling can elicit stronger responses in receivers than signals transmitted via the vascular bundles (Frost et al. 2007; Heil and Silva Bueno 2007; Li and Blande 2017), which may explain the lack of ISR in this experiment.

On the other hand, Tuomi et al. (1988) observed that phenolics only accumulated locally in the leaves of *Betula pubescens* as a result of insect feeding, and not throughout the entire tree canopy. Today, there is increasing evidence that many tree species respond to insect damage at the local rather than the systemic level (Clavijo Mccormick et al. 2014; Mason et al. 2017; Volf et al. 2021). For example, in *Populus nigra* , the release of herbivore‐induced plant volatiles (HIPVs) from damaged leaves was significantly greater than from nearby undamaged leaves (Clavijo Mccormick et al. 2014). Results from a common garden experiment with three broadleaved tree species (*Carpinus betulus, Quercus robur* and *Tilia cordata* ) also suggest that HIPVs are released predominantly from directly affected branches (Volf et al. 2021). Compared to herbaceous plant species, woody plant species are larger, have a more complex canopy architecture, and have longer signaling distances within plant individuals. Furthermore, due to their larger canopy size, trees are more likely to experience localized herbivory than herbaceous species (Volf et al. 2020). These factors may lead to a more localized defense strategy compared to herbaceous species, and the cost–benefit ratio of a systemic response by releasing VOCs to deter herbivores may be less favorable due to the high energy input required to produce VOCs in the whole plant relative to the biomass affected.

The observed chemical variation within the canopy under herbivory is accompanied by large heterogeneities in the microclimate (Lämke and Unsicker 2018), forming a “canopy mosaic,” with different ecological niches for arthropods (Volf et al. 2020). The localization of defenses in the canopy therefore has a direct impact on the distribution of insects at the bottom of the food chain, which in turn can affect the entire trophic network (Lämke and Unsicker 2018; Volf et al. 2020). Furthermore, VOCs released by plants influence atmospheric chemistry by contributing to the formation of ozone and secondary organic aerosols (Griffin et al. 1999; Taipale et al. 2021; Holopainen et al. 2022), which in turn affect cloud formation, albedo, and climate forcing (Shrivastava et al. 2017; Gallo et al. 2024). A process‐based understanding of stress‐induced VOC emissions from trees is thus important to improve our knowledge of the interactions between terrestrial ecosystems and atmospheric processes.

In conclusion, the concept of ISR through vascular within‐plant signaling in trees is challenged by the lack of systemically induced terpenoid emissions from needles and leaves of *P. abies* and *F. sylvatica* in this study. Furthermore, shoot terpenoid emissions were not induced by root‐shoot signaling and *vice versa*. In contrast, terpenoid contents of roots were induced locally and systemically in the *P. abies* root system, as were monoterpene emissions from directly treated roots. This study deepens our understanding of the dynamic production of terpenoids in forest trees under herbivory, and thus of the interactions between plants, insects, and other organisms.

## Author Contributions

M.M., S.H., J.K., J.‐P.S., and C.W. designed the study. A.G. and B.W. performed TD–GC–MS analysis. M.M. analyzed the data and wrote the manuscript with input from all authors.

## Supporting information


**Appendix S1:** Supporting Information.

**Table S1:** Gas exchange parameters.

**Table S2:** Terpenoid emission rates.

**Table S3:** Terpenoid contents.

## Data Availability

The data that support the findings of this study are available from the corresponding author upon reasonable request.
