# Supplementary material for: Localized Response of De Novo Terpenoid Emissions Through the Jasmonate Signaling Cascade in Two Main European Tree Species
Source: Physiol Plant. 2025 Jul 31;177(4):e70432. doi: 10.1111/ppl.70432 (PMC12311559; doi:10.1111/ppl.70432)
Supplement: Supplementary file 1 — Appendix S1: Supporting Information. [file PPL-177-e70432-s003.docx]

## Supporting Information

**Localized response of *de novo* terpenoid emissions through the jasmonate signaling cascade in two main European tree species**
Mirjam Meischner­^*1^, Simon Haberstroh^1^, Jürgen Kreuzwieser^1^, Baris Weber^2^, Andrea Ghirardo^2^, Jörg-Peter Schnitzler^2^, Christiane Werner^1^

¹ Ecosystem Physiology, University of Freiburg, 79110 Freiburg, Germany

^2^ Research Unit Environmental Simulation, Helmholtz Zentrum München, 85764 Neuherberg, Germany

The following Supporting Information is available for this article:

**Fig. S1** Net photosynthesis rate and stomatal conductance of *P. abies* (n=3)

**Fig. S2** Toluene, GLV and methyl salicylate emissions from leaves/needles

**Fig. S3** Ci/Ca ratio of *P. abies*

**Table S1** Gas exchange parameters

**Table S2** Terpenoid emission rates

**Table S2** Terpenoid contents

**Methods S1** Calculation of VOC fluxes and gas exchange parameters

**Methods S2** TD-GC-MS analysis

**Fig. S1** Net photosynthesis rate (A) and stomatal conductance (Gs) of *P. abies* (n=3)

**
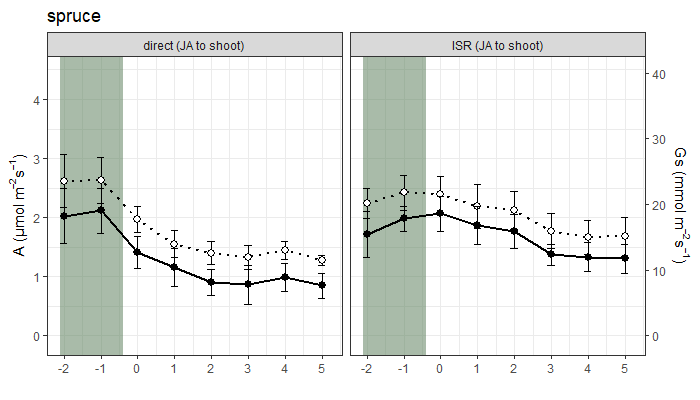
**

**
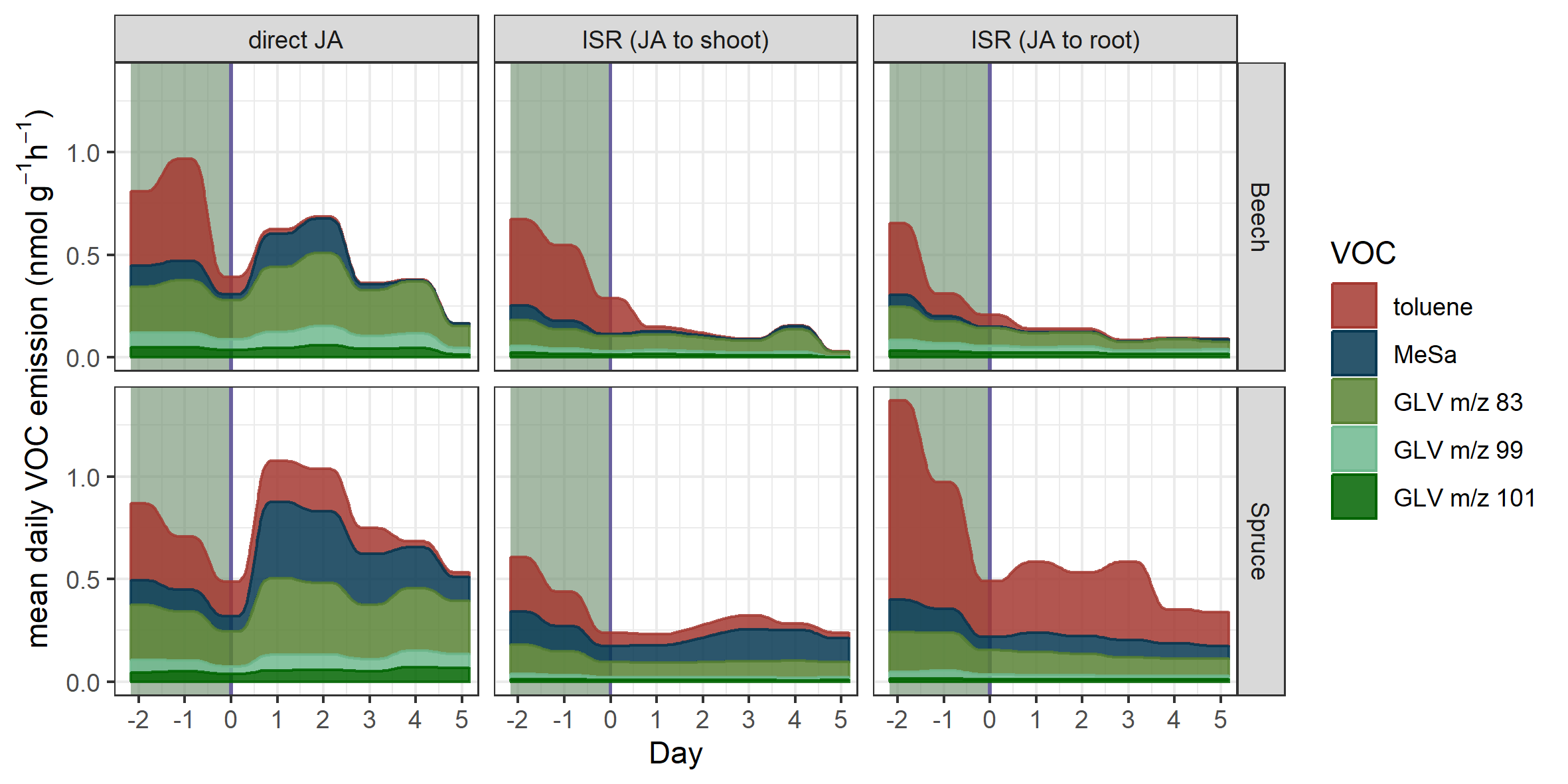
Fig. S2** Toluene, methyl salicylate and green leaf volatile (GLV) emissions from leaves/needles

**Fig. S3** Ratio of intercellular and ambient CO2 concentrations (Ci/Ca) in needles of *P. abies*. Daly means ± standard errors (n=6) are shown. The green background indicates the control phase before jasmonic acid application on day zero*.*


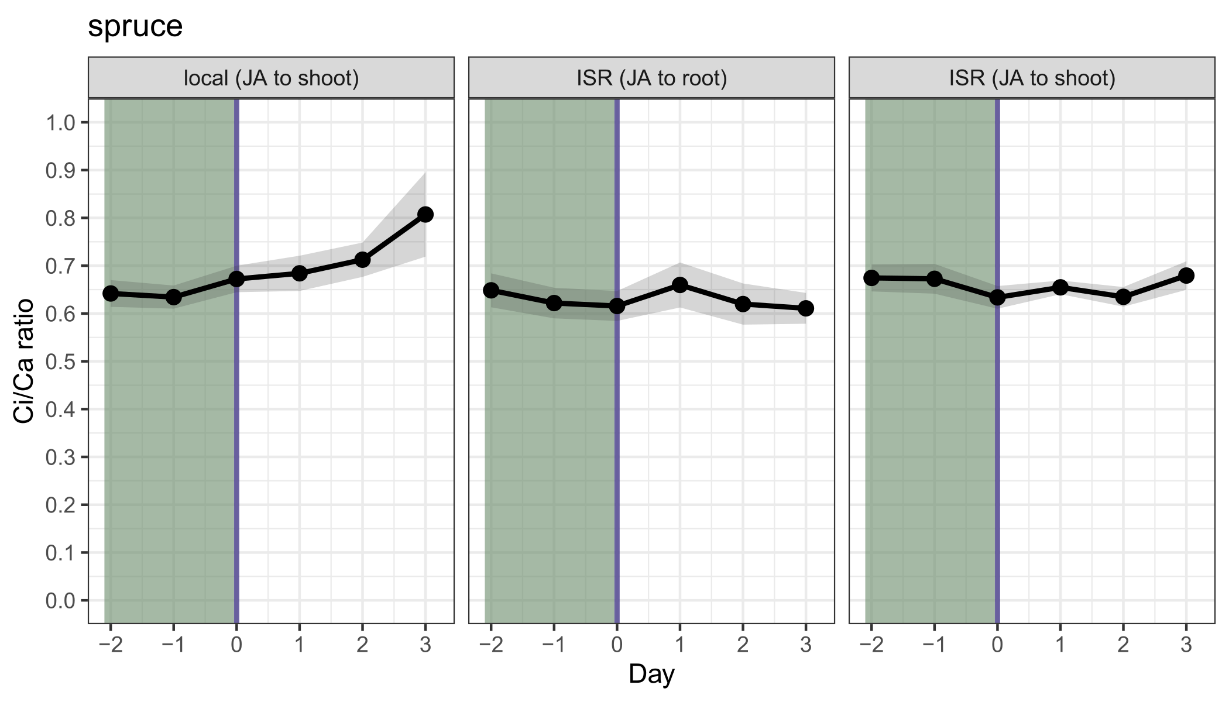


**Methods S1** Calculation of VOC fluxes and gas exchange parameters

VOC fluxes

To retrieve VOC fluxes and gas exchange parameters, the molar flow u (mol s^−1^) through the cuvettes was determined with Equation (1)

| $u=\frac{V}{t}*\frac{p}{R*T}$ | ($SEQ Equation \backslash* ARABIC$ $1$) |
| --- | --- |

where V is the gas volume (m^3^), t is time (s), p is air pressure (Pa), R is the molar gas constant (J mol^−1^ K^−1^) and T is temperature (K).

To retrieve VOC fluxes e (mol g^−1^ s−^1^) Equation (2) was used:

| $e=\frac{u}{w}*\left( VOCo-VOCa \right)$ | ($SEQ Equation \backslash* ARABIC$ $2$) |
| --- | --- |

where w is the fresh weight of roots or leaves (g) and VOCo and VOCa are the concentrations of VOCs (mol mol^-1^) at the cuvette outlet and inlet, respectively.

Root respiration rates

Analogously, root respiration rate Rs (mol g-1 s-1) was calculated with Equation (3):

| $Rs=\frac{u}{w}*\left( r\_co-r\_ca \right)$ | ($SEQ Equation \backslash* ARABIC$ $3$) |
| --- | --- |

where w is the fresh weight of roots and r_co and r_ca are the concentrations of CO_2_ (mol mol^-1^) at the outlet and inlet of the root cuvette, respectively.

Transpiration and net photosynthesis rates

First the transpiration rate E (mol m^-2^ s^-1^) was determined according to von Caemmerer & Farquhar (1981) with Equation (4):

| $E=\frac{u}{s}*\frac{wo-wi}{1-wo}$ | ($SEQ Equation \backslash* ARABIC$ $4$) |
| --- | --- |

where s is leaf area (m^2^) and wo and wi are the concentrations of H_2_O (mol mol^-1^) at the outlet and inlet of the cuvette, respectively.

Finally, net photosynthesis rate (mol m^-2^ s^-1^) was calculated using Equation (5):

| $A=\frac{u}{s}*\left( \frac{1-wi}{1-wo} \right)*\left( ca-co \right)-E*ci$ | ($SEQ Equation \backslash* ARABIC$ $5$) |
| --- | --- |

Stomatal conductance

The absolute humidity inside the cuvette AH_c_ (g m^-3^) based on the measured concentration of H_2_O (mol mol^-1^) at the cuvette inlet was calculated with Equation (6):

| ${AH}_{c}=\frac{wi*m}{V_{m}}$ | ($SEQ Equation \backslash* ARABIC$ $6$) |
| --- | --- |

where m is the molecular weight of water (= 18.01528 g mol^-1^) and V_m_ the molar volume of an ideal gas at [standard temperature and pressure](https://en.wikipedia.org/wiki/Standard_temperature_and_pressure) (22.4·10^−3^ m^3^ mol^-1^).

The absolute humidity inside the leaf AH_l_ was determined based on the measured temperature according to Steubing & Fangmeier (1992). First the saturation vapor pressure e (kPa) was calculated with equation (7):

| $P= 101,325\exp\left( 13,3185t-1,976t^{2}-0,6445t^{3}-0,1229t^{4} \right)$ | ($SEQ Equation \backslash* ARABIC$ $7$) |
| --- | --- |

where t is defined as $t= 1-(Ts/T)$ and Ts as vapour temperature at standard air pressure (=373.15 K).

The vapor pressure was then converted into absolute humidity (g m^-3^) using Equation (8) according to Lösch (2001):

| ${AH}_{l}=\left( \frac{2.17}{T} \right)*P*1000$ | ($SEQ Equation \backslash* ARABIC$ $8$) |
| --- | --- |

The absolute water vapour pressure deficit (VPD) (g m^-3^) is the difference between AH_l_ and AH_c_ (equation 9):

| $VPD={AH}_{l}-{AH}_{c}$ | ($SEQ Equation \backslash* ARABIC$ $9$) |
| --- | --- |

Finally, stomatal conductance for water vapour Gs (mol m^-2^ s-^1^) is calculated with Equation (10)

| $Gs=\frac{\frac{E*m}{VPD}}{Vm}$ | ($SEQ Equation \backslash* ARABIC$ $10$) |
| --- | --- |

Ratio of intercellular and ambient CO_2_ concentrations

The stomatal conductance for CO_2_ G_CO2_ (mol m^-2^ s-^1^) was calculated from Gs under consideration of the diffusion coefficient for CO_2_ with Equation (11)

| $G_{{CO}_{2}}=\frac{Gs}{1.56}$ | ($SEQ Equation \backslash* ARABIC$ $11$) |
| --- | --- |

The intracellular concentration of CO_2_ C_i_ (mol mol^-1^) was then calculated with Equation (12)according to von Caemmerer & Farquhar (1981)

| $C_{i}=\frac{\left( G_{{CO}_{2}}-\frac{E}{2} \right)*C_{a}-A}{\left( G_{{CO}_{2}}+\frac{E}{2} \right)}$ | ($SEQ Equation \backslash* ARABIC$ $12$) |
| --- | --- |

Finally, the ratio between intercellular and ambient CO_2_ concentrations is C_i_/C_a_.

**References**

**von Caemmerer S, Farquhar GD**. **1981**. Some relationships between the biochemistry of photosynthesis and the gas exchange of leaves. *Planta* **153**: 376–387.

**Lösch R**. **2001**. *Wasserhaushalt der Pflanzen*. Wiebelsheim: Quelle & Meyer.

**Steubing L, Fangmeier A**. **1992**. *Pflanzenökologisches Praktikum: Gelände- und Laborpraktikum der terrestrischen Pflanzenökologie*. Stuttgart: Ulmer.

**Methods S2: TD-GC-MS analysis**

Per sample, one µL of hexane extract (plant/needle or root extract) containing 859.3 pmol µL^-1^ of δ-2-carene (Merck Chemicals GmbH, Germany) as an internal standard was analyzed by thermo-desorption gas-chromatography mass-spectrometry (TD-GC–MS; TD, Gerstel; GC, 7890A and MS, 5975C both from Agilent Technologies), run similarly to previous analyses (Ghirardo *et al.*, 2012, 2016, 2020). The sample was injected directly into a glass insert placed in the glass tube of the TD unit (Gerstel GmbH, Mülheim an der Ruhr, Germany) kept at 30°C, and evaporated to 250 °C at a rate of 280°C min^-1^, and held for 5.00 min. The headspace of the glass vial was transferred to a Tenax TA liner (re-focusing) in the cryo-injection system (CIS) maintained at 20°C with a vent flow of 60 ml min^-1^ and at a vent pressure of 10 psi. The transfer line between the TDU and CIS was maintained at 250°C. After 0.42 min of the CIS-transfer, terpenes were desorbed from the CIS-liner by increasing the temperature in the CIS to 270°C at a rate of 12°C s^-1^ and holding for 4.00 min, using a purge flow of 100 ml min^-1^ and switching to spitless mode after 0.5 min and back to split mode after 2 min. Chromatographic separation was achieved using a 5% phenyl 95% dimethyl arylene siloxane capillary column (60 m × 250 μm × 0.25 μm J&W DB-5MS + 10 m DG, Agilent Technologies) and a constant flow of helium (purity of 5.0) at 1 mL min^-1^. The temperature program of the GC oven started at 40°C, increased to 150°C at a rate of 10°C min^-1^, then increased to 175°C at 80°C min^-1^, following a ramp from 175°C to 190°C at 5°C min^-1^, then from 190°C to 250°C at 80°C min^-1^, and finally to 300°C at 100°C min^-1^ and held for 6 min. Ionization and detection were performed by electron impact (70 eV) and a quadrupole mass-spectrometer detector (MSD). The MS source and quadrupole temperatures were set to 230 and 150 °C, respectively. Data were acquired in both scan mode (range of 35-300 m/z, 4 cycles, threshold of 150 counts) and single ion mode (SIM), monitoring the characteristic terpene ions at m/z 68, 92, 93, 95, 120, 121, 136, 152, 154, 161, and 204 between 10-50 ms dwell time. Each sample was analyzed in duplicate, and averages of the two technical replicates were used for further analysis.

**References**

**Ghirardo A, Heller W, Fladung M, Schnitzler J-P, Schroeder H**. **2012**. Function of defensive volatiles in pedunculate oak *(Quercus robur*) is tricked by the moth *Tortrix viridana*. *Plant, Cell & Environment* **35**: 2192–2207.

**Ghirardo A, Lindstein F, Koch K, Buegger F, Schloter M, Albert A, Michelsen A, Winkler JB, Schnitzler J-P, Rinnan R**. **2020**. Origin of volatile organic compound emissions from subarctic tundra under global warming. *Global Change Biology* **26**: 1908–1925.

**Ghirardo A, Xie J, Zheng X, Wang Y, Grote R, Block K, Wildt J, Mentel T, Kiendler-Scharr A, Hallquist M, *et al.*** **2016**. Urban stress-induced biogenic VOC emissions and SOA-forming potentials in Beijing. *Atmospheric Chemistry and Physics* **16**: 2901–2920.
